# Queer in Chem: Q&A with Professor Polly Arnold

**DOI:** 10.1038/s42004-023-00971-w

**Published:** 2023-09-30

## Abstract

Polly Arnold is a Professor of chemistry at the University of California, Berkeley and Director of the Chemical Sciences Division at Lawrence Berkeley National Laboratory in the US. Polly’s research focuses on exploratory synthetic chemistry. Such knowledge underpins the discovery of catalysts and our understanding of the behavior of nuclear waste.

**Figure:**
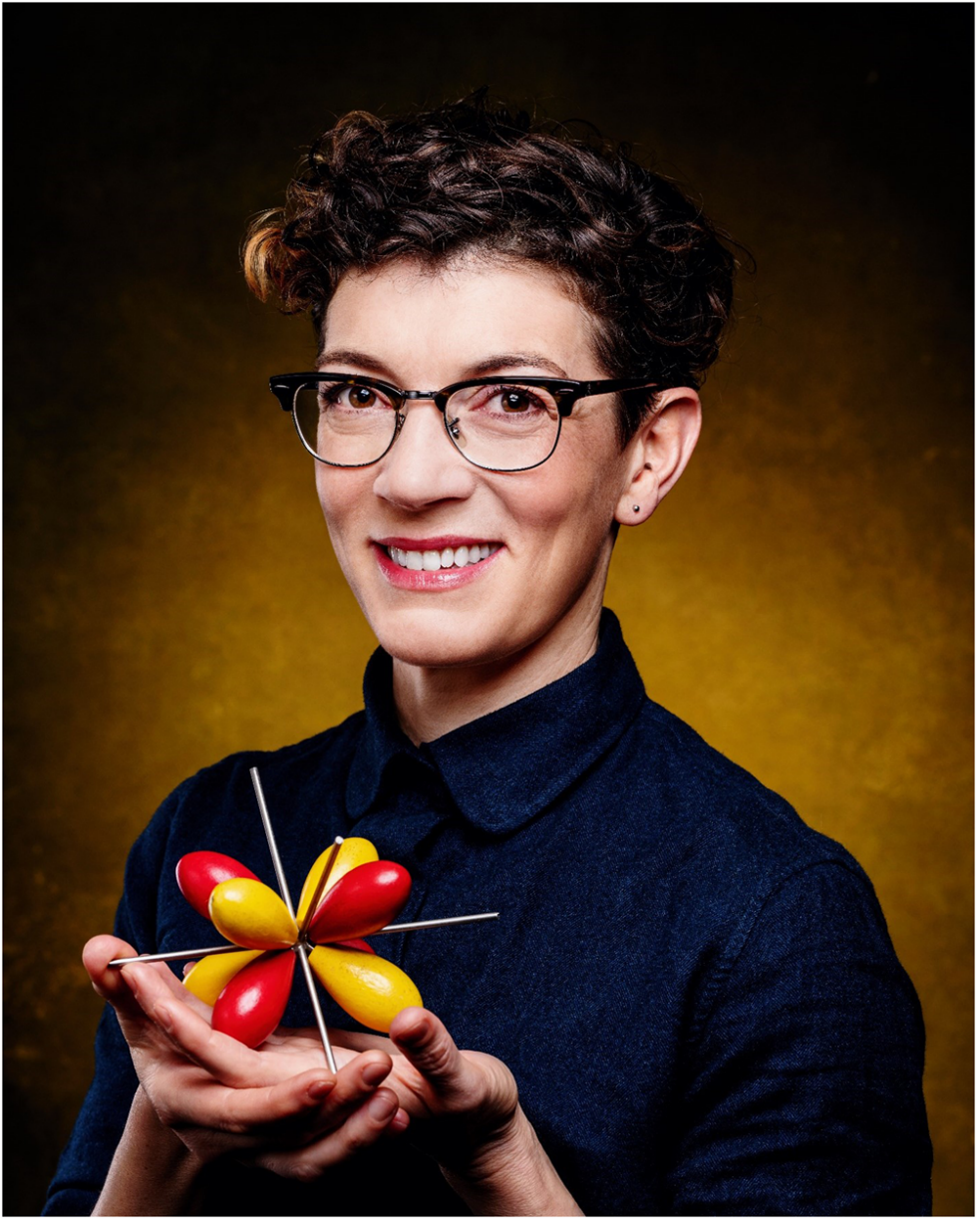
Portrait courtesy of the RSE’s ‘Women in Science in Scotland’ exhibition, taken by Ian Georgeson

Polly Arnold advises government and industry and appears regularly in mainstream media to discuss the importance and benefits of diversity in science, technology, engineering, and mathematics (STEM). She was awarded the Rosalind Franklin Award in 2012 for her scientific achievements and her role in promoting women in STEM. That same year, she was elected a Fellow of the Royal Society of Edinburgh. In 2015, Polly was awarded a UK Research Council Suffrage Science award, and in 2017, the Lord Kelvin Prize, Scotland’s senior research prize in the physical sciences. She was appointed Officer of the Order of the British Empire in 2017. In 2018, she was the first woman to be awarded the Royal Society of Chemistry Sir Geoffrey Wilkinson award for her work on transuranic organometallic chemistry. She was elected a Fellow of the Royal Society in 2018 for substantial contributions to the improvement of natural knowledge.

Why did you choose to be a scientist?

I have always loved problem solving and hated writing essays.

What scientific development are you currently most excited about?

I’m delighted that lanthanide-dependent microbes are being discovered everywhere^1–3^ – in the oceans, in soils, and in the phyllospheres of plants – not just in the extremophiles that were reported with such excitement a few years ago. It makes so much more sense to me that nature would choose to use the whole periodic table. Equality of opportunity for all the elements.

What direction do you think your research field should go in?

I think we need to be working more closely with our computational chemistry collaborators, and it needs to be easier for them to be able to calculate much larger systems much more quickly. Many systems need to describe the multiconfigurational ground state of a molecule to accurately describe catalytic reactions. Chemists are using larger ligands and more metal centers to improve and discover new reactions, and it would be wonderful to be able to design and develop these complicated systems alongside, and with the predictive capabilities of theory.

Why do you think it is important to feel comfortable enough to bring your whole self to work?

Because constantly self-censoring is emotionally exhausting. Each small effort to avoid naming or gendering the person you share your adventures with adds up. This is mental energy that I would rather be spending on chemistry.

How can individual scientists support and celebrate their LGBTQ+ colleagues?

By being allies in front of others, and otherwise, by not making a big deal about anyone’s gender or sexuality. A couple of years ago, I saw one minority woman PhD student interrupt a senior establishment figure in a meeting to politely call out their misnaming of another minority early career scientist, which took so much courage; and again, a lot of mental energy. When I see senior people who are members of majority communities correcting mistakes or calling out inappropriate behavior, if anything, it increases their social capital, and it takes the pressure away from the minoritized populations to be the ones always on watch.

What action(s) do you feel employers in chemical research should take to make a difference for LGBTQ+ scientists?

Employers must recognize that international travel can be frightening and dangerous. I am lucky to live in a part of a country that is extremely accepting. I want international researchers who are out and proud here to be able to return safely to their home country without fear of prejudice. Further, fear of safety or discrimination should never have to be a factor in the decision to travel to a foreign placement or conference.

*This interview was conducted by the editors of Communications Chemistry*.
